# A novel saponin liposomes based on the couplet medicines of *Platycodon grandiflorum–Glycyrrhiza uralensis* for targeting lung cancer

**DOI:** 10.1080/10717544.2022.2112997

**Published:** 2022-08-23

**Authors:** Chunjing Guo, Yanguo Su, Hui Wang, Min Cao, Ningning Diao, Zhongxin Liu, Daquan Chen, Ming Kong

**Affiliations:** a College of Marine Life Science, Ocean University of China, Qingdao266003, P.R. China; b School of Pharmacy, Yantai University, Yantai264005, P.R. China; c Gynecology Department, Affiliated hospital of Weifang Medical University, Weifang261053, P.R. China

**Keywords:** Couplet medicines, liposomes, synergistic therapy, platycodin

## Abstract

Liposomes have been widely used for targeted drug delivery, but the disadvantages caused by cholesterol limit the application of conventional liposomes in cancer treatment. The compatibility basis of couplet medicines and the compatibility principle of the traditional Chinese medicine principle of ‘monarch, minister, assistant and guide’ are the important theoretical basis of Chinese medicine in the treatment of tumor and the important method to solve the problem of high toxicity. In this study, the active ingredients of the couplet medicines *Platycodon grandiflorum* and *Glycyrrhiza uralensis* were innovatively utilized, and glycyrrhizic acid (GA) was encapsulated in liposomes constructed by mixing saponin and lecithin, and cholesterol was replaced by platycodin and ginsenoside to construct saponin liposomes (RP-lipo) for the drug delivery system of Chinese medicine. Compared with conventional liposomes, PR-lipo@GA has no significant difference in morphological characteristics and drug release behavior, and also shows stronger targeting of lung cancer cells and anti-tumor ability in vitro, which may be related to the pharmacological properties of saponins themselves. Thus, PR-lipo@GA not only innovatively challenges the status of cholesterol as a liposome component, but also provides another innovative potential system with multiple functions for the clinical application of TCM couplet medicines.

## Introduction

Lung cancer is one of the malignancies with the highest incidence and mortality rates worldwide, and non-small cell lung cancer (NSCLC) accounts for up to 85% of these cancers (Bonanno et al., 2011; Passaro et al., 2022). Currently, the main treatment strategies for lung cancer patients include surgery, chemotherapy and radiotherapy. Although chemotherapy is the preferred option (Legha et al., 2015; Meng et al., 2022), treatment efficacy is hampered by the development of drug resistance, unfavorable pharmacokinetics and insufficient accumulation of current therapeutic agents intratumorally. Meanwhile, conventional drug formulations are difficult to deliver to tumor tissues and therefore frequent drug administration may increase the incidence of toxic side effects.

Recently, countless studies have reported that the combination of traditional Chinese medicine (TCM) can effectively improve the tumor condition and has the effect of increasing efficiency and reducing toxicity (Chen et al., 2021; Jiang et al., 2016; Lu et al., 2022). The couplet medicines are the bridge from a single Chinese medicine to a compound prescription, and the rules and mechanisms of the couplet medicines are one of the foundations of modern research on Chinese medicine compound prescriptions. Among the herbal formulas handed down to this day, the couplet medicines of *Platycodon grandiflorum–Glycyrrhiza uralensis* are often used in combination as the channel ushering drug for the lungs, which is beneficial for the treatment of lung diseases. The results of modern studies have also shown that the use of *P. grandiflorum* and *G. uralensis* can effectively increase the aggregation concentration of roxithromycin and doxorubicin in the lung (Yang et al., 2012; Li et al. 2005). Licorice has the effects of invigorating the spleen and benefiting the lung, clearing away heat and detoxifying. Glycyrrhetinic acid is a bioactive ingredient extracted from licorice and it also has anti-inflammatory and antiviral activities. In addition, it has toxic effects on a variety of tumor cells. At present, glycyrrhetic acid has been used in the targeted therapy of liver cancer based on the receptors of glycyrrhizic acid (GA) present on liver cancer cells. Studies have shown that GA can block the division and replication cycle of cancer cells in the G0/G1 phase, and can also induce mitochondria-dependent apoptosis in A549 lung cancer cells. Potential anti-cancer mechanisms are all GA can be used for the treatment of lung cancer and provide support (Zhu et al., 2015; Cai et al., 2016; Luo et al., 2021).

Nanoparticle-based drug delivery systems are widely used in cancer therapeutic research for their excellent drug delivery ability in tumors and their controlled drug release characteristics (Amin et al., 2021; Capolla, 2021; Saadat et al., 2021). Among the many nanocarriers, liposomes have been extensively noticed for their excellent biocompatibility and high loading capacity for drugs with different physicochemical properties (Li et al., 2020; Narendra et al., 2020; Zhou et al., 2020). The ability to prolong the long circulation of drug delivery systems in the blood and avoid elimination by the reticuloendothelial system (RES) is often achieved by surface modification of polyethylene glycol (PEG) to deliver drugs to tumor tissues. Although polyethylene glycolylation could prolong the circulation time of liposomes in the blood, excessive PEG exposure can lead to the production of anti-PEG IgG, which induces immune responses and RES phagocytosis (Hu et al., 2009; Su et al., 2018). Therefore, the clinical application of polyethylene glycolylated nanocarriers remains a challenge.

The construction of multifunctional liposomes and their safety has been a hot topic of research since cholesterol is one of the components of liposomes, however, numerous studies have found that cholesterol has a series of side effects in the machine and has been questioned in clinical practice (Szebeni et al., 2000; Moein Moghimi et al., 2006). There are various substances with similar structure to cholesterol in nature, such as sterols (Yang et al., 2013; Albuquerque et al., 2018; Monpara et al., 2018) and saponins, which are active substances widely found in plants and mushrooms. Among them, saponins are abundant and mostly have good anticancer, anti-inflammatory, immunomodulatory and other medicinal values. The saponins are used as lipid membrane modulating substances to replace cholesterol wrapped in liposomes to obtain new functional liposomes with pharmacological activity more promising applications (Lu et al., 2018; Li et al., 2019). Ginsenosides are the active ingredients extracted from ginseng belonging to sterol compounds, and there are many types, such as Rb1, Rb2, Rg1, Rg3, Rh2 etc. Different ginsenosides have different inhibitory effects on tumor cells (Ratan et al., 2021; Jung et al., 2022). It has been reported that paclitaxel-ginsenoside liposomes prepared by ginsenoside substitution for cholesterol have similar properties to cholesterol liposomes in terms of particle size and encapsulation rate, and have better long-circulation effects as well as synergistic anti-gastric cancer efficacy (Hong et al., 2019). In addition, ginsenosides have been shown to be substrates of glucose-related transporter proteins, which are highly expressed in most types of tumor cells, and thus ginsenosides could assist liposome accumulation at tumor sites by interacting with glucose transporter proteins (Chang et al., 2007).

*P. grandiflorum*, known as the boat of the lungs, is often used to treat lung diseases. *P. grandiflorum* saponins are the active ingredients of *P. grandiflorum* as the key ingredient for introducing medicine into the lungs. *P. grandiflorum* saponins have anti-inflammatory and immune regulation (Shen et al., 2019). The solubilizing effect determined by the surface activity and the effect on cell membrane permeability of platycodin, as the active component of *P. grandiflorum*, contribute to drug aggregation in the lung may be the intrinsic basis for its channel affinity (Zhao et al., 2015; Zhao et al., 2015).

**Figure 1.**
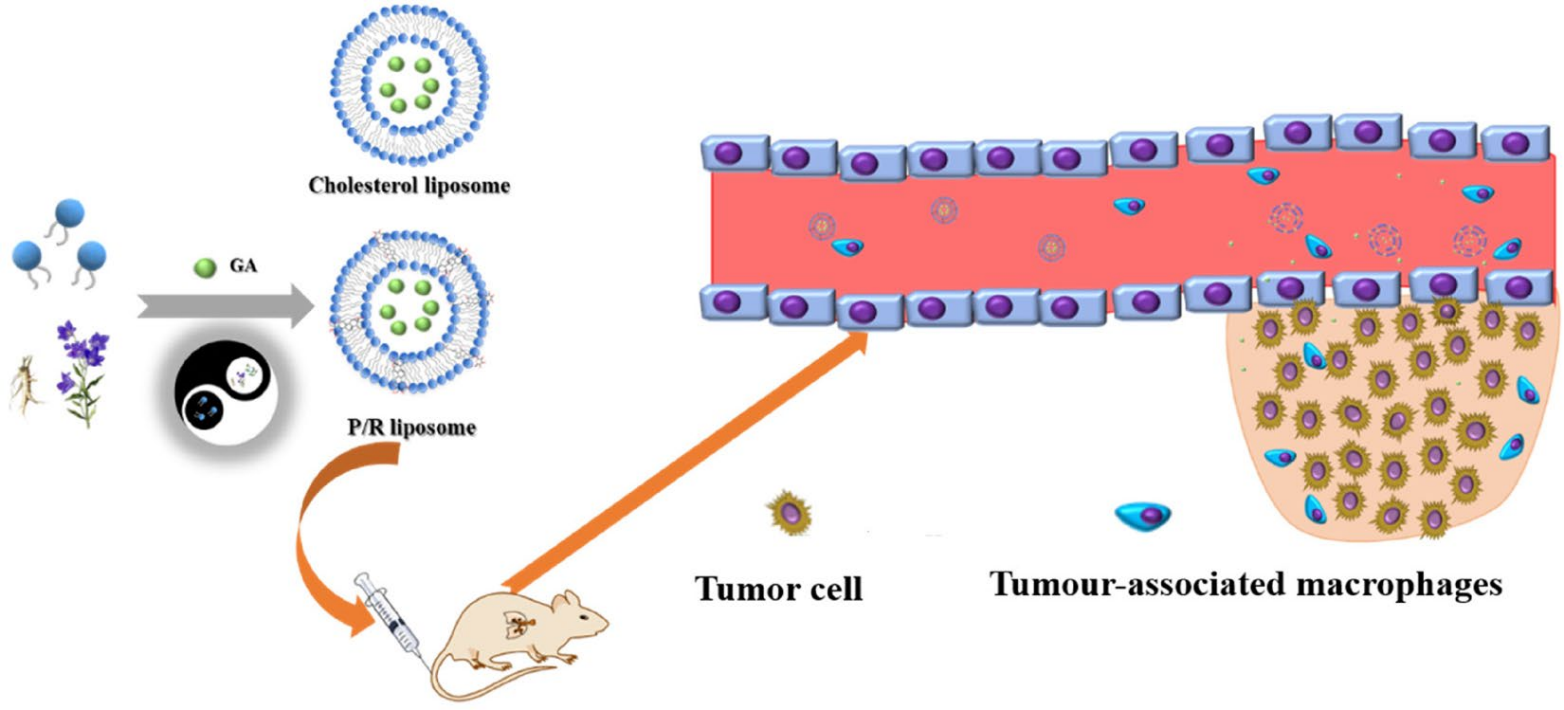
The rational design of a multifunctional PR-lipo for targeted tumor therapy.

According to the couplet medicines of TCM and the ‘monarch, minister, assistant and guide’ combination, this study has creatively interpreted it using nanodelivery system. Through the couplet medicines of *P. grandiflorum* and *G. uralensis* pair as the entry point, we used GA as the model drug and acted as the monarch drug. Ginsenoside and platycodin replace cholesterol and form mixed saponin liposomes with phospholipids as drug delivery systems, in which ginsenoside is the minister drug, phospholipid is the assistant drug, and platycodin is the envoy drug, combining the traditional principles of TCM compounding with modern targeted nanotechnology to achieve a synergistic therapeutic effect of two birds with one stone (Figure 1).

## Materials and methods

### Materials

Lecithin and cholesterol were purchased from Aladdin (China). Ginsenoside, platycodin and glycyrrhizic acid were obtained from Shanghai Yuanye Biotechnology Co (Shanghai, China). Coumarin 6 fluorescent dye was purchased from Adamas (Shanghai, China). MTT kits and apoptosis kits were purchased from Shanghai Beyotime Biotechnology Co (Shanghai, China). The chemical reagents used in this experiment were purchased from Aladdin (China). The dialysis bag was purchased from Beijing Bailingwei Company (Beijing, China).

### Preparation and characterization of liposomes

Preparation of each group of liposomes was done by film dispersion method. Cholesterol liposomes (C-lipo@GA) were prepared from lecithin to cholesterol (10:3, mass ratio), ginsenoside liposomes (R-lipo@GA) were prepared from lecithin to ginsenoside (10:3, mass ratio) and mixed saponin liposomes (PR-lipo@GA) were prepared from lecithin to ginsenoside and platycodin (10:2:1, mass ratio). Briefly, phospholipids and cholesterol/saponins are dissolved in an organic solvent (chloroform:ethanol = 1:1). Homogeneous lipid films were formed by rotary evaporator at 45 °C. The lipid films were hydrated with 2 mL of saline at 45 °C for 30 min, followed by sonication at 300 W, 20 Hz for 5 min under ice bath conditions to obtain a blank liposome solution.

The drug-laden liposomes were prepared by the same procedure as earlier, with a 10:1 mass ratio of lecithin to GA. The prepared drug-laden liposomes were passed through 220-nm microporous filter membrane to obtain homogeneous liposome solutions and then placed in a refrigerator at 4 °C. Subsequently, the particle size and zeta potential of each group of liposomes were measured by dynamic light scattering (DLS) with a Delsa Nano C (Beckman Coulter, USA). The drug-laden liposomes were appropriately diluted, stained with 1% mass fraction of phosphotungstic acid and then morphologically observed under transmission electron microscope (TEM, H-600; Hitachi, Tokyo, Japan).

### The drug content and release profile in vitro

The drug content in liposomes was determined by liquid chromatography (HPLC, Agilent 1260, USA). Methanol and 1% glacial acetic acid aqueous solution (89:11, vol%) was chosen as the mobile phase with detection at 250 nm. Then, the drug encapsulation efficiency (EE, %) and loading efficiency (LC, %) were calculated separately according to the following equations.

(1) $$\mathrm{EE}(\%)=\frac{\text{weight of loaded GA}}{\text{weight of GA in feed}}\times 100\%$$

(2) $$\mathrm{LC}(\%)=\frac{\text{weight of loaded GA }}{\text{weight of GA loaded NPs}}\times 100\%$$

The in vitro drug release behavior of C-lipo@GA, P-lipo@GA and RP-lipo@GA was investigated by using dialysis method to examine whether there is any difference in drug release from different formulations. Briefly, 2 mL of drug-laden liposomes were measured in a dialysis bag (MW: 2000 DA), placed in 45 mL of phosphate buffered salt solution (PBS) release medium. As for the 37 °C thermostatic shakers, 1 mL of sample was taken at a predetermined time point and supplemented with an equal amount of blank medium. After microporous membrane filtration, the peak areas were obtained using HPLC and the cumulative release of the samples at each time point was calculated according to the following equation.

(3) $$\text{Cumulative drug release}(\%)=\frac{M_{t}}{M_{0}}\times 100\%$$

where *M_t_* is the weight of drug released at time *t* and M_0_ is the weight of loaded drug. The release of the experiments was performed in triplicate.

### The stability of liposomes

The stability of C-lipo@GA, P-lipo@GA and RP-lipo@GA were examined separately under the same in vitro environment. Briefly, C-lipo@GA, P-lipo@GA and RP-lipo@GA were incubated with PBS containing 10% fetal bovine serum for 48 h in an in vitro simulated blood environment, and the degree of change in particle size was measured by DLS.

### Cell culture

RAW264.7, HUVECs and A549 cells were purchased from ATCC. Cells were cultured in dulbecco’s modified eagle medium (DMEM) high-glucose medium containing 10% fetal bovine serum (FBS) and 1% penicillin–streptomycin at 37 °C in a 5% CO_2_ incubator.

### The biocompatibility of liposomes

In this study, the biocompatibility of liposomes was investigated at the cellular and blood levels, respectively. The effects of blank liposomes (RP-lipo) on the cell viability of normal cells were investigated by 3-(4,5-Dimethyl-2-Thiazolyl)-2,5-Diphenyl Tetrazolium Bromide (MTT) method. We selected HUVECs and RAW264.7 cells as normal cell models, and RP-lipo was incubated with them for 24 h, respectively, to detect the toxic effects of different concentrations of blank vector on vascular endothelial cells and immune cells.

Considering that saponins may cause hemolysis, we evaluated the hemolytic behavior of P-lipo@GA and RP-lipo@GA, respectively. Briefly, after blood sampling from mouse orbits, blood was centrifuged for 10 min at 4 °C, 800*g*, and the lower red blood cells were collected and resuspended with isotonic PBS to obtain red blood cell suspensions, which were co-incubated with P-lipo@GA and RP-lipo@GA saline solutions, and saline and deionized water were set as negative and positive control groups, respectively, and left for 4 h at room temperature, and the hemolysis of each group was observed by centrifugation at 800*g* for 10 min under room temperature. The supernatant of each group was collected, and the absorbance of each group was measured at 540 nm by multifunctional enzyme marker, and the corresponding hemolysis rate was calculated by analysis.

### Cellular uptake and localization in vitro

To investigate the uptake ability of the prepared mixed saponin liposomes by different cells, so as to evaluate its targeting effect on tumor cells, coumarin 6(C6) was used as a fluorescent probe to study the uptake of free C6, C-lipo@C6, P-lipo@C6, RP-lipo@C6 in RAW264.7 and A549 cells. RAW264.7 cells were inoculated in 12-well plates at 2.0 × 10^5^ cells/well, and after incubation overnight, cells were co-incubated with media containing the same concentration (200 ng/mL) of C6, C-lipo@C6, P-lipo@C6 and RP-lipo@C6 for 1 h, 2 h, 3 h and 4 h, respectively. Subsequently, the medium was washed three times with pre-cooled PBS, fixed and stained cells with 4’,6-diamidino-2-phenylindole (DAPI), and after washing three times with pre-chilled PBS, the uptake status of different groups of preparations by RAW264.7 cells was observed in fluorescent inverted microscope. In vitro targeting studies of A549 cells were performed as described earlier.

### Cytotoxicity assay

The cytotoxicity of different preparation groups to A549 cells was determined by MTT method. Briefly, A549 cells were co-incubated with different concentrations of free GA, C-lipo@GA, P-lipo@GA, RP-lipo@GA for 48 h, followed by MTT assay. IC_50_ was calculated using GraphPad Prism software.

### Apoptosis assay

The apoptotic status of A549 cells was detected by apoptosis and necrosis assay kit using Hoechst 33342 and double staining with propidium iodide (PI), which cannot stain normal cells with intact cell membranes. In contrast, for necrotic cells, the integrity of their cell membranes was lost and staining was possible. A549 cells were inoculated in 24-well plates, incubated overnight and then co-incubated with the same drug concentration (50 µg/mL) of free GA, C-lipo@GA, P-lipo@GA and RP-lipo@GA for 12 h. The cells were then stained and the staining effect was observed using an inverted fluorescence microscope.

### Statistical analysis

The obtained results are expressed as mean ± SD and all statistical analyses were obtained with GraphPad Prism version 8.0 software (GraphPad, USA). One-way ANOVA and two-way ANOVA were used for statistical analyses. Significance levels were set at *p < .05, **p < .01 and ***p < .001. Expression of all fluorescence intensities in the experiments was further calculated by Image J software.

## Results and discussion

### Characterization of saponin liposomes

**Figure 2.**
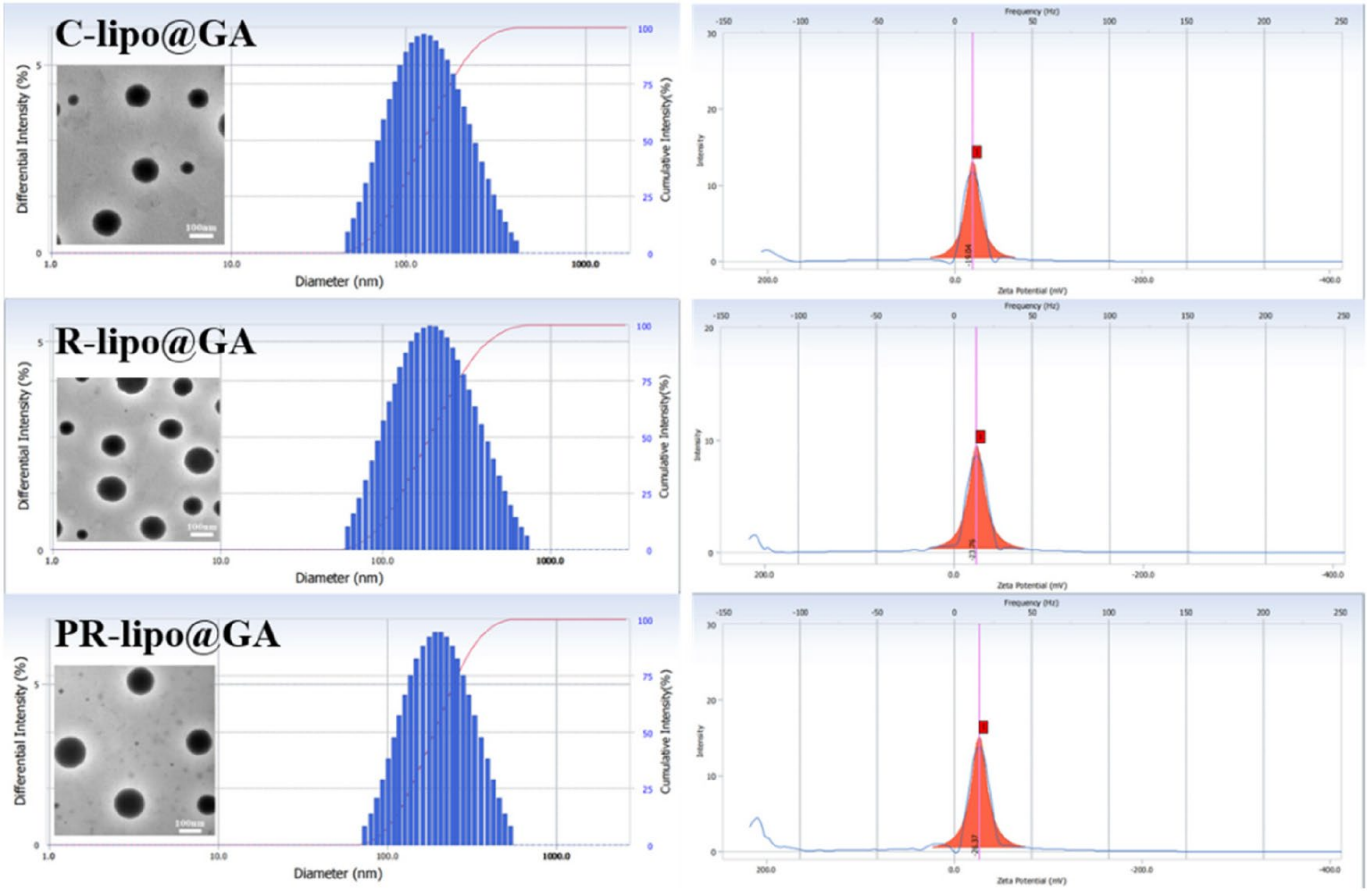
Dynamic light scattering and transmission electron microscopy images of C-lipo@GA, R-lipo@GA and PR-lipo@GA (scale bar = 100 nm).

**Figure 3.**
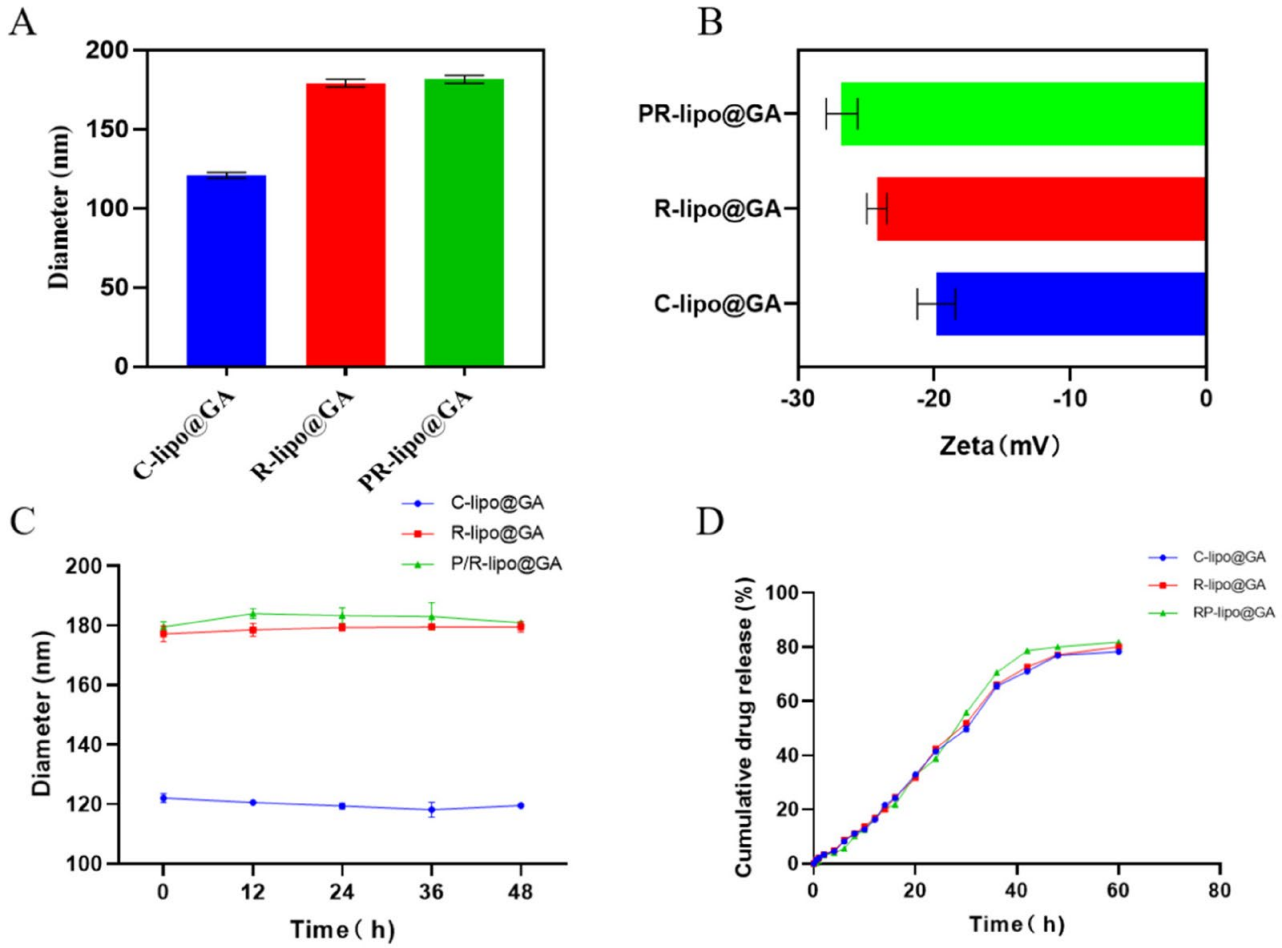
Characterization of saponin liposomes. (A) Particle size variation of different formulations of liposomes. (B) Variation of zeta potential for different formulations of liposomes. (C) Change in size of different liposomal formulations stored at 4 °C. (D) Study of in vitro drug release behavior of liposomes. Data are shown as the mean ± SD (*n* = 3).

The preparation process of saponin liposome and normal cholesterol liposome is the same, and they are both obtained by hydration through the film dispersion method. As shown in the Figure 2(A) both R-lipo@GA and PR-lipo@GA have spherical shapes with smooth surfaces, which are basically consistent with cholesterol liposomes. Therefore, substitution of saponins did not significantly alter the morphology of liposomes. As shown in Table 1, the particle sizes of R-lipo@GA and PR-lipo@GA are 179.07 ± 2.40 nm and 181.63 ± 2.53 nm (Figure 3(A)), respectively, which are larger than the average particle size of C-lipo@GA (120.90 ± 1.87 nm). This particle size change is similar to EE%, which may be due to the high encapsulation efficiency of the drug in the saponin liposome structure, resulting in a corresponding increase in the particle size of the nanoparticles. Meanwhile, the zeta potential of R-lipo@GA and PR-lipo@GA is more negative than that of C-lipo@GA (Figure 3(B)). This change in physical and chemical properties is worth thinking about. Generally speaking, when the absolute value of Zeta potential is higher, it means that the system has excellent stability, and the negative value of surface potential is more obvious, it is easier to achieve long-term circulation effect, and it is not easy to be taken up by phagocytes. In summary, we speculate that the unique physicochemical properties of saponins may affect the ordering and fluidity of phospholipid molecules, thereby changing the size and surface state of liposomes.

**Table 1.** Characterization of GA-loaded liposomes (*n* = 3; mean ± standard deviation).

	Size	PDI	ZP (mV)	EE (%)	LE (%)
C-lipo@GA	120.90 ± 1.87	0.16 ± 0.020	–20.02 ± 1.26	67.16 ± 1.48	4.14 ± 0.09
R-lipo@GA	179 ± 2.40	0.21 ± 0.017	–24.21 ± 0.74	81.02 ± 1.40	4.99 ± 0.09
PR-lipo@GA	181.63 ± 2.53	0.17 ± 0.003	–26.78 ± 1.15	86.62 ± 0.61	5.33 ± 0.04

The stability of liposomes is one of the inherent conditions for evaluating whether they can release drugs in vivo and reduce drug toxicity and side effects. As shown in the Figure 3(C,D), C-lipo@GA, R-lipo@GA and PR-lipo@GA were able to maintain stable particle size in the simulated blood environment in vitro, verifying that the saponins possess similar properties to cholesterol and can replace cholesterol in the original liposome prescription and still maintain the properties of liposome encapsulation rate, release rate and stability.

### The biocompatibility of saponin liposomes

**Figure 4.**
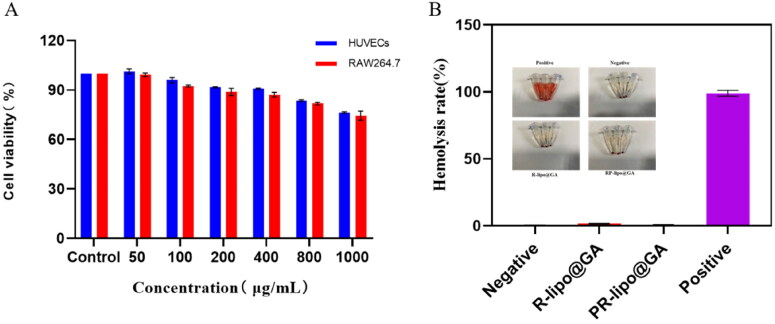
Biocompatibility evaluation of PR-lipo. (A) Cytotoxicity of blank vectors against RAW264.7 and HUVECs cells. (B) Hemolytic properties of saponin liposomes.

This study investigated the biocompatibility of PR-lipo at cellular and blood levels. HUVECs as well as RAW264.7 cells were used as a model to examine the effect of blank liposomes on the activity of normal cells. As shown in the Figure 4(A), the cellular activity remained around 80% at the highest concentration treatment for 24 h, indicating its good biocompatibility at the cellular level. In addition, as shown in the Figure 4(B), neither R-lipo@GA nor PR-lipo@GA had obvious hemolytic effect, and the hemolysis rate was less than 2%, indicating that saponin liposomes did not cause hemolysis. The constructed saponin liposomes possess remarkable biocompatibility.

### Cellular uptake and targeting capabilities

**Figure 5.**
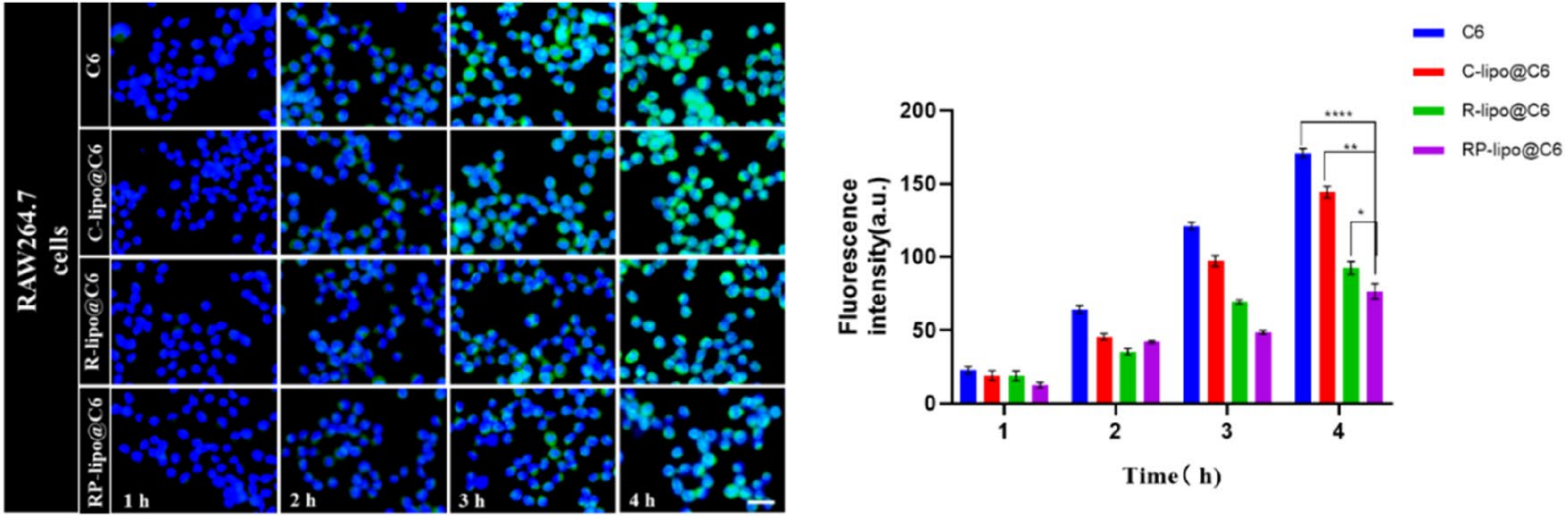
Cellular escape capacity of different liposomes after co-incubation with RAW264.7 cells (scale bar = 50 µm).

In order for the drug to effectively kill tumor cells, the nanoparticles must have certain cellular escape ability to avoid being phagocytosed by immune cells, delivered to the lesion in the blood circulation and taken up by tumor cells, thus releasing the drug to effectively treat cancer. In this study, RAW264.7 was used as a cellular model to investigate the cellular escape ability of different preparations. As shown in the Figure 5, there was a time-dependent fluorescence intensity of RAW264.7 after co-incubation with free C6, C-lipo@C6, P-lipo@C6 and RP-lipo@C6 in all groups. However, RP-lipo@C6 showed a relatively low intracellular green fluorescence signal compared to the other preparations, indicating that it was least taken up by RAW264.7 cells.

**Figure 6.**
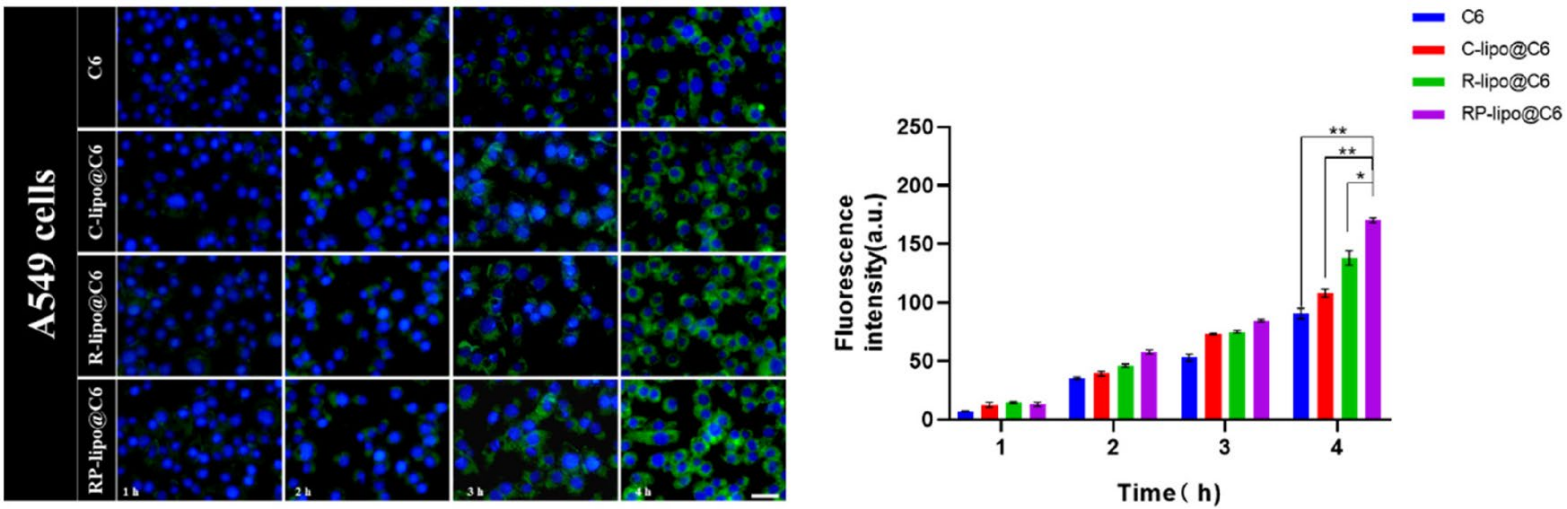
In vitro targeting ability of different liposomes on A549 cells (scale bar = 50 µm).

The targeting ability of each group of agents on lung cancer cells was examined by co-incubating A549 cells with free C6, C-lipo@C6, P-lipo@C6 and RP-lipo@C6. Interestingly, we found that P-lipo@C6 and RP-lipo@C6 had relatively high intracellular fluorescence intensity, with RP-lipo@C6 having the highest fluorescence intensity contrary to the cell escape results (Figure 6), indicating that it was taken up by A549 in the highest amount.

In summary, the saponin liposomes constructed in this study possessed excellent cell escape ability as well as tumor cell targeting ability, and we speculate that it may be that saponin acts as a substrate for glucose-related transporter protein in tumor cells and thus effectively targets A549 cells, however, the targeting effect of RP-lipo@C6 was more pronounced compared to P-lipo@C6, which may be a synergistic effect of platycodon saponins and ginsenoside targeting effect.

### In vitro antitumor activity of GA-loaded saponin liposomes

**Figure 7.**
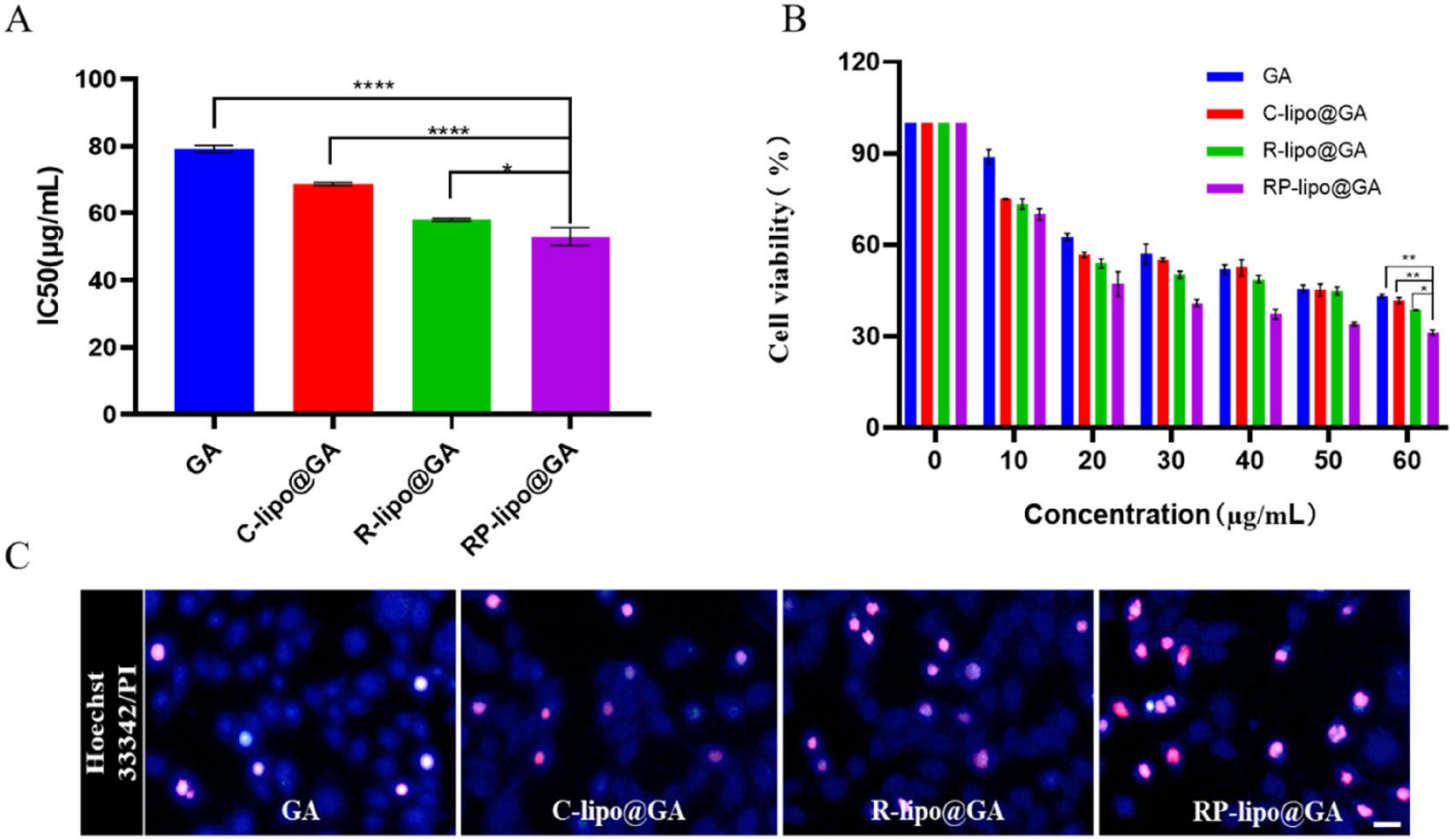
In vitro anticancer activities of GA-loaded saponin liposomes. A and B: IC_50_ values and Cytotoxicity of free GA and different types of GA-loaded liposomes in A549 cells. C: Apoptotic status of A549 cells treated with different preparation groups (Red color represents apoptotic cells, scale bar = 50 µm).

To detect the cytotoxicity of RP-lipo@GA on tumor cells, we performed MTT and apoptosis assays using A549 cells in vitro. As shown in Figure 7(A,B), free GA, C-lipo@GA, P-lipo@GA and RP-lipo@GA all showed a concentration-dependent inhibition of tumor cell growth. In contrast, RP-lipo@GA has the strongest inhibitory ability, and its half inhibitory concentration (IC_50_) is only 0.62 for GA, 0.73 for C-lipo@GA and 0.85 for P-lipo@GA. Therefore, we can conclude that the admixture of platycodin and ginsenoside significantly promoted the antitumor activity compared to conventional liposomes.

Apoptosis was detected by Hoechst 33342/PI double staining, as shown in the Figure 7(C), RP-lipo@GA caused apoptosis in A549 cells up to about 60%. The apoptosis in the free GA group was less than 10%, while C-lipo@GA and P-lipo@GA increased the total apoptosis to about 10% and 30%, respectively. The P-lipo@GA was due to the synergistic effect between ginsenosides and GA resulting in increased apoptosis, and RP-lipo@GA was due to the synergistic promotion of the main active components of the couplet medicines of *P. grandiflorum* and *G. uralensis*, which together with ginsenosides increased antitumor activity. Thus, the mixed saponin is not only a membrane material in this formulation, it is also an adjuvant drug. It embodies the drug delivery strategy of killing two birds with one stone by using both drugs and adjuvants.

## Conclusion

In summary, a new multifunctional liposome delivery system was constructed, based on the concept of couplet medicines and ‘monarch, minister, assistant and guide’ combination, through the couplet medicines of *P. grandiflorum* and *G. uralensis* as the entry point, and GA as the model drug. Ginsenoside and platycodin were used to replace cholesterol, it reflects the advantages of traditional Chinese medicine in anticancer treatment. At the same time, ginsenoside and platycodon grandiflorum saponin replace cholesterol, and phospholipid to construct mixed saponin liposomes RP-lipo@GA as a drug delivery system, can have basically the same morphology as conventional liposomes, which provides a strong basis for the preparation of mixed saponin instead of cholesterol liposomes. Mixed saponins are not only used as adjuvant chemotherapy, but also as a membrane stabilizer with long blood circulation time. More importantly, they have excellent cell escape ability and targeting ability to lung cancer cells. These promising results suggest that liposomes with mixed saponins innovatively challenge the irreplaceable status of cholesterol as a liposome component and can prove to be an effective drug delivery system for curing cancer in the future.

## References

[CIT0001] Albuquerque HMT, Santos CMM, Silva AMS. (2018). Cholesterol-based compounds: recent advances in synthesis and applications. Molecules. 4:116. 10.3390/molecules24010116 PMC6337470 30597999

[CIT0002] Amin MU, Ali S, Ali MY, et al. (2021). Enhanced efficacy and drug delivery with lipid coated mesoporous silica nanoparticles in cancer therapy. Eur J Pharm Biopharm. 165:31–40. 33962002 10.1016/j.ejpb.2021.04.020

[CIT0003] Bonanno L, Favaretto A, Rugge M, et al. (2011). Role of genotyping in non-small cell lung cancer treatment. Drugs. 71:2231–46. 10.2165/11597700-000000000-00000 22085382

[CIT0004] Cai Y, Xu Y, Chan HF, et al. (2016). Glycyrrhetinic acid mediated drug delivery carriers for hepatocellular carcinoma therapy. Mol Pharm. 13:699–709. 26808002 10.1021/acs.molpharmaceut.5b00677

[CIT0005] Capolla SJP. (2021). Nanoparticles-based oligonucleotides delivery in cancer: role of zebrafish as animal model. Pharmaceutics. 13:1106. 34452067 10.3390/pharmaceutics13081106 PMC8400075

[CIT0006] Chang TC, Huang SF, Yang TC, et al. (2007). Effect of ginsenosides on glucose uptake in human Caco-2 cells is mediated through altered Na+/glucose cotransporter 1 expression. J Agric Food Chem. 55:1993–8. 17269785 10.1021/jf062714k

[CIT0007] Chen Y, Sun Y, Zhao Q, et al. (2021). Shenmai injection enhances cisplatin-induced apoptosis through regulation of Mfn2-dependent mitochondrial dynamics in lung adenocarcinoma A549/DDP cells. Cancer Drug Resist. 4(4):1047–60. 35582383 10.20517/cdr.2021.94 PMC8992451

[CIT0008] Hong C, Wang D, Liang J, et al. (2019). Novel ginsenoside-based multifunctional liposomal delivery system for combination therapy of gastric cancer. Theranostics. 9:4437–49. 31285771 10.7150/thno.34953 PMC6599661

[CIT0009] Hu X, Subramanyam M, Lerner M, et al. (2009). Pharmacokinetic and pharmacodynamic profile of PEGylated interferon beta-1a in healthy volunteers: results from two clinical studies. Br J Clin Pharmacol. 79:514–22.

[CIT0010] Jiang Y, Liu LS, Shen LP, et al. (2016). Traditional Chinese medicine treatment as maintenance therapy in advanced non-small-cell lung cancer: a randomized controlled trial. Complement Ther Med. 24:55–62. 26860802 10.1016/j.ctim.2015.12.006

[CIT0011] Jung D-H, Nahar J, Mathiyalagan R, et al. (2022). Focused review on molecular signalling mechanisms of ginsenosides on anti-lung cancer and anti-inflammatory activities. Anticancer Agents Med Chem. 22:1875–5992. 10.2174/1871520622666220321091022 35319393

[CIT0012] Legha SS, Muggia FM, Carter SKJC. (2015). Adjuvant chemotherapy in lung cancer. Rev Prospects. 39:1415–24. 10.1002/1097-0142(197704)39:4<1415::aid-cncr2820390410>3.0.co;2-o 192430

[CIT0013] Li N, Mai Y, Liu Q, et al. (2020). Docetaxel-loaded d-α-tocopheryl polyethylene glycol-1000 succinate liposomes improve lung cancer ­chemotherapy and reverse multidrug resistance. Drug Deliv Transl Res. 11:131–41. 10.1007/s13346-020-00720-9 32052357

[CIT0014] Li R, Zhang L, Li Z, et al. (2019). Characterization and absorption kinetics of a novel multifunctional nanoliposome stabilized by sea cucumber saponins instead of cholesterol. J Agric Food Chem. 68: 642–651. 31830780 10.1021/acs.jafc.9b06460

[CIT0015] Li Y-L, Lu S-M, Jian M. (2005). Effect of Chinese herb *Platycodon grandiflorum* on Roxithromycin concentration of lung tissue. J Tradit Chin Vet Med. 2005: 3–6.

[CIT0016] Lu L, Ding Y, Zhang Y, et al. (2018). Antibody-modified liposomes for tumor-targeting delivery of timosaponin AIII. Int J Nanomed. 13:1927–44. 10.2147/IJN.S153107 PMC5880182 29636610

[CIT0017] Lu L, Dong J, Gong W, et al. (2022). Advances in basic research of traditional Chinese medicine herbal formulae in the treatment of non-small cell lung cancer. Tradit Med Modern Med. 1–11.

[CIT0018] Luo Y-H, Wang C, Xu W-T, et al. (2021). 18β-Glycyrrhetinic acid has anti-Cancer effects via inducing apoptosis and G2/M cell cycle arrest, and inhibiting migration of A549 lung cancer cells. Onco Targets Ther. 14:5131–44. 34712051 10.2147/OTT.S322852 PMC8548027

[CIT0019] Meng LF, Huang JF, Luo PH, et al. (2022). The efficacy and safety of immune checkpoint inhibitor plus chemotherapy in patients with advanced non-small-cell lung cancer: a meta-analysis. Invest New Drugs. 40:810–17. 35412172 10.1007/s10637-022-01232-8

[CIT0020] Moein Moghimi S, Hamad I, Bünger R, et al. (2006). Activation of the human complement system by cholesterol-rich and PEGylated liposomes-modulation of cholesterol-rich liposome-mediated complement activation by elevated serum LDL and HDL levels. J Liposome Res. 16:167–74. 16952871 10.1080/08982100600848801

[CIT0021] Monpara J, Kanthou C, Tozer GM, et al. (2018). Rational design of cholesterol derivative for improved stability of paclitaxel cationic liposomes. Pharm Res. 35:90. 29520495 10.1007/s11095-018-2367-8

[CIT0022] Narendra, Mehata AK, Viswanadh MK, et al. (2020). Formulation and in vitro evaluation of upconversion nanoparticle-loaded liposomes for brain cancer. Ther Deliv. 11:557–71. 32867624 10.4155/tde-2020-0070

[CIT0023] Passaro A, Brahmer J, Antonia S, et al. (2022). Managing resistance to immune checkpoint inhibitors in lung cancer: treatment and novel strategies. J Clin Oncol. 40: 598–610. 34985992 10.1200/JCO.21.01845

[CIT0024] Ratan ZA, Haidere MF, Hong YH, et al. (2021). Pharmacological potential of ginseng and its major component ginsenosides. J Ginseng Res. 45:199–210. 33841000 10.1016/j.jgr.2020.02.004 PMC8020288

[CIT0025] Saadat M, Mostafaei F, Mahdinloo S, et al. (2021). Drug delivery of pH-sensitive nanoparticles into the liver cancer cells. J Drug Deliv Sci Technol. 63:102557.

[CIT0026] Shen F, Wu W, Zhang M, et al. (2019). Micro-PET imaging demonstrates 3-O-β-d-glucopyranosyl platycodigenin as an effective metabolite affects permeability of cell membrane and improves dosimetry of [18F]-phillygenin in lung tissue. Front Pharmacol. 10:1020. 31572193 10.3389/fphar.2019.01020 PMC6753856

[CIT0027] Su Y, Wang L, Liang K, et al. (2018). The accelerated blood clearance phenomenon of PEGylated nanoemulsion upon cross administration with nanoemulsions modified with polyglycerin. Asian J Pharm Sci. 13:44–53. 32104377 10.1016/j.ajps.2017.07.003 PMC7032119

[CIT0028] Szebeni J, Baranyi L, Savay S, et al. (2000). Liposome-induced pulmonary hypertension: properties and mechanism of a complement-mediated. Am J Physiol Heart Circ Physiol. 279:H1319. 10993799 10.1152/ajpheart.2000.279.3.H1319

[CIT0029] Yang B, Geng SY, Wang JY. (2013). Physical stability of cholesterol derivatives combined with liposomes and their in vitro behavior. Annu Int Conf IEEE Eng Med Biol Soc. 2013:4114–17. 24110637 10.1109/EMBC.2013.6610450

[CIT0030] Yang EJ, Ju SM, Ku HY, et al. (2012). Isoliquiritigenin isolated from *Glycyrrhiza uralensis* protects neuronal cells against glutamate-induced mitochondrial dysfunction. Biochem Biophys Res Commun. 421(4):658–64. 22538371 10.1016/j.bbrc.2012.04.053

[CIT0031] Zhao R, Chen M, Jiang Z, et al. (2015). Platycodin-D induced autophagy in non-small cell lung cancer cells via PI3K/Akt/mTOR and MAPK signaling pathways. J Cancer. 6:623–31. 26078792 10.7150/jca.11291 PMC4466411

[CIT0032] Zhao RL, Zhang X, Chen MJ, et al. (2015). Research progress in anti-tumor mechanisms of platycodin-D. J Infrom Tradit Chin Med. 2015:126–129.

[CIT0033] Zhou L, Lu R, Liu Q, et al. (2020). Two branched fructose modification improves tumor targeting delivery of liposomes to breast cancer in intro and in vivo. J Drug Deliv Sci Technol. 61:102312.

[CIT0034] Zhu J, Chen M, Chen N, et al. (2015). Glycyrrhetinic acid induces G1-phase cell cycle arrest in human non-small cell lung cancer cells through endoplasmic reticulum stress pathway. Int J Oncol. 46:981–8. 25573651 10.3892/ijo.2015.2819 PMC4324580

